# Synthesis and Characterization of a Linear [Mn_3_(O_2_CMe)_4_(py)_8_]^2+^ Complex

**DOI:** 10.1155/2010/932569

**Published:** 2010-05-30

**Authors:** Eleni E. Moushi, Christos Kizas, Vassilios Nastopoulos, Anastasios J. Tasiopoulos

**Affiliations:** ^1^Department of Chemistry, University of Cyprus, 1678 Nicosia, Cyprus; ^2^Department of Chemistry, University of Patras, 26500 Patras, Greece

## Abstract

Two new compounds that consist of the linear trinuclear manganese(II) cation [Mn_3_(O_2_CMe)_4_(py)_8_]^2+^ cocrystallizing with different counteranions (I_3_^−^, [**1**]; ClO_4_^−^, [**2**]) are reported. Complex **1** was prepared from the reaction of [Mn(O_2_CMe)_2_] · 4H_2_O with I_2_ in MeCO_2_H/py, whereas complex **2** was isolated from the reaction of [Mn_3_O(O_2_CMe)_6_(py)_3_] · py with [Mn(ClO_4_)_2_] · 6H_2_O in MeCN/py. The crystal structures of both compounds were determined by single crystal X-ray crystallography. Magnetic susceptibility studies that were performed in microcrystalline powder of **1** in the 2–300 K range revealed the presence of antiferromagnetic exchange interactions that resulted in an S = 5/2 ground spin state.

## 1. Introduction

Oligonunuclear Mn carboxylate clusters have attracted significant interest since they have been located in the active site of metalloenzymes [1] and also often have interesting and sometimes novel magnetic properties [2]. Undoubtedly, the most well-known oligonuclear cluster that appears in biological systems is the tetranuclear Mn complex that is present in the active site of photosystem II and is responsible for the light driven oxidation of water to molecular dioxygen [3–7]. Other Mn compounds observed in the active sites of metalloenzymes involve mononuclear (e.g., in Mn-superoxide dismutases) [8] and dinuclear (e.g., in Mn-catalases) complexes [8, 9]. In all those compounds, the ligation of the Mn ions is provided mainly by O- and N-donor atoms from the various aminoacid residues present in the metalloproteins. In order to prepare functional and structural models of the Mn compounds that are present in metalloenzymes, efforts have been centered on the synthesis and study of manganese carboxylate complexes with various chelating N-donor ligands, such as 2,2′-bipyridine (bpy) [10–13], 1,10-phenanthroline (phen) [10, 14–16], and 2-(2-pyridyl)benzimidazole [17]. As a result a plethora of dinuclear, trinuclear and tetranuclear manganese compounds containing carboxylato groups or/and nitrogen-donor ligands have been prepared and characterized [4, 7, 10–30]. Such complexes are of significant interest not only as potential functional and structural models of the metal clusters present in Mn-containing metalloenzymes but also as precursors for the isolation of new model compounds. In particular, trinuclear Mn compounds have attracted significant attention since they appear as discrete metal clusters with various topologies including linear [10–26], triangular [27], V-shaped [28], and so forth, clusters and also as building blocks in multidimensional coordination polymers [29]. Linear trinuclear manganese (II) clusters with various molecular formulas such as [Mn_3_(O_2_CR)_6_(L)_2_] [10–20] and [Mn_3_(O_2_CR)_4_(L′)_2_] [21–23] have been prepared with several types of carboxylates, bidentate (L), and tridentate or tetradentate (L′) chelates and also terminal ligands. 

Herein, we report the synthesis, structural characterization, and magnetic properties of a new linear manganese(II) cation, [Mn_3_(O_2_CMe)_4_(py)_8_]^2+^ which cocrystallizes with two different counteranions (I_3_^−^, [**1**] and ClO_4_^−^, [**2**]). The cation of **1** and **2** represents the first linear trinuclear Mn^II^ unit that contains only carboxylate and pyridine ligands and a rare example of a linear Mn^II^_3_ cluster that is stabilized with carboxylate and terminal ligands without containing any polydentate chelates [30].

## 2. Experimental 

### 2.1. Materials

All manipulations were performed under aerobic conditions using materials (reagent grade) and solvents as received; water was distilled in-house. [Mn_3_O(O_2_CMe)_6_(py)_3_] · py was prepared as described elsewhere [27]. *Warning: Although we encountered no problems, appropriate care should be taken in the use of the potentially explosives perchlorate anion.*

### 2.2. Syntheses of Compounds

#### 2.2.1. [Mn_3_(O_2_CMe)_4_(py)_8_](I_3_)_2_ [**1**]

Solid I_2_ (2.07 g, 8.16 mmol) was added to the yellowish solution of [Mn(O_2_CMe)_2_] · 4H_2_O (2.00 g, 8.16 mmol) in MeCOOH/py (10/20 mL). The resulting red-brown solution was left under magnetic stirring for ~45 minutes, filtered off and the filtrate was left undisturbed at room temperature. After a few weeks, dark brown crystals of **1** suitable for X-ray crystallography were formed. The crystals were collected by filtration, washed with MeCOOH/py (5/10 mL) and dried in vacuum. The yield was ~60% based on total Mn content. A sample for crystallography was maintained in contact with the mother liquor to prevent the loss of interstitial solvent. *Anal. Calc.* for C_48_H_52_Mn_3_N_8_O_8_I_6_ [**1**]: C, 32.11; H, 2.92; N, 6.24. Found: C 31.89; H 2.79; N 6.10%. IR data (KBr pellet, cm^−1^): $\tilde{\nu }=3435$ (m), 3059 (m), 1599 (s), 1580 (s), 1564 (s, br), 1483 (m), 1441 (s, br), 1350 (m), 1215 (m), 1151 (m), 1067 (m), 1038 (m), 1005 (m), 752 (m), 700 (s), 683 (m), 650 (m), 629 (m).

#### 2.2.2. [Mn_3_(O_2_CMe)_4_(py)_8_](ClO_4_)_2_ [**2**]

**Method A**

To a solution of [Mn_3_O(O_2_CMe)_6_(py)_3_] · py (0.294 g, 0.345 mmol) in MeCN/py (10/2 mL) was added Mn(ClO_4_)_2_ · 6H_2_O (0.125 g, 0.345 mmol) and pdH_2_ (0.10 mL, 0.105 g, 1.38 mmol) and the mixture was left under magnetic stirring for ~30 minutes. The resulting dark red-brown slurry was filtered off and the dark red-brown filtrate was left undisturbed at room temperature. After few weeks yellow crystals appeared, suitable for X-ray structural determination. The crystals were isolated by filtration, washed with a copious amount of MeCN/py, and dried in vacuum; yield, ~20% based on total ClO_4_^−^ content. A sample for crystallography was maintained in contact with the mother liquor to prevent the loss of interstitial solvent. *Anal. Calc.* for C_48_H_52_Mn_3_N_8_O_16_Cl_2_ [**2**]: C, 46.77; H, 4.25; N, 9.09. Found: C 46.63; H 4.09; N 8.95%.

**Method B**

Method A was repeated in a mixture of MeCN/py (10/4 mL) without using H_2_pd. The yield was ~9% based on on total ClO_4_^−^ content.

### 2.3. X-Ray Crystallography

Data were collected on an Oxford-Diffraction Xcalibur diffractometer, equipped with a CCD area detector and a graphite monochromator utilizing Mo-K*α* radiation (*λ* = 0.71073 Å). Suitable crystals were attached to glass fibers using paratone-N oil and transferred to a goniostat where they were cooled for data collection. Unit cell dimensions were determined and refined by using 12271 (3.07 ≤ *θ* ≤ 30.27°) and 5746 (3.06 ≤ *θ* ≤ 30.29°) reflections for **1** and **2,** respectively. Empirical absorption corrections (multiscan based on symmetry-related measurements) were applied using CrysAlis RED software [31]. The structures were solved by direct methods using SIR92 [32], and refined on *F* ^2^ using full-matrix least squares with SHELXL97 [33]. Software packages used: CrysAlis CCD [31] for data collection, CrysAlis RED [31] for cell refinement and data reduction, WINGX for geometric calculations [34], and DIAMOND [35] and MERCURY [36] for molecular graphics. The non-H atoms were treated anisotropically, whereas the hydrogen atoms were placed in calculated, ideal positions and refined as riding on their respective carbon atoms. Unit cell data and structure refinement details are listed in Table 1.

### 2.4. Physical Measurements

Elemental analyses were performed by the in-house facilities of the Chemistry Department, University of Cyprus. IR spectra were recorded on KBr pellets in the 4000–400 cm^−1^ range using a Shimadzu Prestige-21 spectrometer. Variable-temperature DC magnetic susceptibility data down to 1.80 K were collected on a Quantum Design MPMS-XL SQUID magnetometer equipped with a 70 kG (7 T) DC magnet. Diamagnetic corrections were applied to the observed paramagnetic susceptibilities using Pascal's constants. Samples were embedded in solid eicosane, unless otherwise stated, to prevent torquing.

## 3. Results and Discussions

### 3.1. Syntheses

Both complexes were prepared serendipitously during our investigations on two different synthetic methods. The first one involved the use of iodine as an oxidizing agent in various reactions of [Mn(O_2_CMe)_2_] · 4H_2_O, while the second one included the employment of 1,3-propanediol (pdH_2_) in reactions with [Mn_3_O(O_2_CMe)_6_(py)_3_] · py.

One of the most successful strategies to polynuclear Mn clusters has been the oxidation of a Mn^2+^ starting material with the use of various oxidizing agents, often in the presence of a chelating ligand. Several oxidants have been employed for this purpose such as MnO_4_^−^, Ce^IV^, peroxides, bromate, and iodine to form high-oxidation state Mn species [4, 7, 28]. Although the use of iodine as oxidant in Mn cluster chemistry has been reported in the past [4, 7], the oxidation of Mn^2+^ salts from iodine under various conditions is a rather unexplored synthetic method. Compound **1** was prepared during our investigations on reactions of Mn(O_2_CMe)_2_ · 4H_2_O with iodine in MeCOOH/pyridine. A large amount of MeCOOH was used in order to avoid the formation of various Mn oxides/hydroxides that precipitate at basic conditions. Thus, the reaction of [Mn(O_2_CMe)_2_] · 4H_2_O with solid I_2_ in a 1 : 1 ratio in MeCOOH/py (10/20 mL) resulted in the formation of dark brown crystals of **1** in ~60% yield. The formation of **1 **is summarized in (1):

(1) $$\begin{array}{cc} & 3\left[\text{Mn}{\left({\text{O}}_{2}\text{CMe}\right)}_{2}\right]+3{\text{I}}_{2}+{\text{H}}_{2}\text{O}+8\text{py}\\ & \to \left[\text{M}{\text{n}}_{3}{\left({\text{O}}_{2}\text{CMe}\right)}_{4}{\left(\text{py}\right)}_{8}\right]{\left({\text{I}}_{3}\right)}_{2}+2\text{MeCOOH}+1/2{\text{O}}_{2}. \end{array}\;$$

Despite the presence of an oxidant (I_2_) in the reaction mixture, the final product (compound **1**) contains only Mn^2+^ ions. We believe that species that contain Mn ions in higher oxidation states are also formed but are quite soluble and thus do not precipitate from the reaction solution. 

Another synthetic method to new polynuclear Mn clusters employed recently by our group involves the use of aliphatic diols such as pdH_2_ in Mn cluster chemistry. These studies have resulted in a number of new polynuclear clusters and coordination polymers with coordinated pdH_2_ ligands [37–40]. Many of these compounds were isolated from reactions that were involving the use of [Mn_3_O(O_2_CMe)_6_(py)_3_] · py as a starting material [37, 38]. These studies, apart from compounds that contain coordinated pdH_2_ ligands, have also resulted in complexes that do not include the diol in their asymmetric unit, with **2** being one of the members of this family. Thus, compound **2 **was initially prepared from the reaction of [Mn_3_O(O_2_CMe)_6_(py)_3_] · py with Mn(ClO_4_)_2_ · 6H_2_O in the presence of pdH_2_ in a 1 : 1 : 4 ratio in MeCN/py (10/2 mL) in 20% yield. When the identity of **2 **was established and known that it contained neither coordinated nor lattice pdH_2_/pd^2-^ ligands, the reaction resulted in the formation of **2 **was repeated without including pdH_2_ in the reaction mixture. This reaction gave a few crystals of **2**. Various modifications were applied in this reaction in order to optimize its yield. Finally, the larger yield (achieved when no pdH_2_ was included in the reaction mixture) was ~9% and obtained when an extra amount of pyridine (4 more mL) was added to the reaction solution. The exact role of pdH_2_ in the assembly of **2** and how its use results in larger reaction yield still remain unidentified.

### 3.2. Description of the Structures

**Figure 1:**
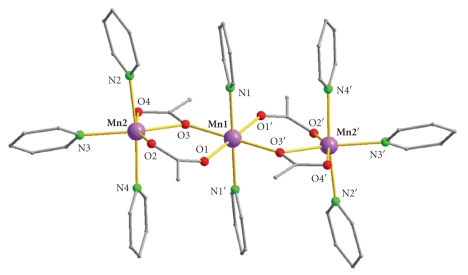
A partially labeled plot of the cation of **1**. Color code: Mn, purple; O, red; N, green; C, grey. H atoms are omitted for clarity.

The molecular structure of complex **1** is presented in Figure 1 and selected interatomic distances and angles for **1 **are listed in Table 2. Bond valence sum (BVS) calculations for the metal ions of **1** and **2** are given in Table 3. The crystal structures of **1** and **2** present a striking similarity with the main difference between them being their counter-ions and thus only that of **1** will be described here.

Compound **1 **crystallizes in the monoclinic P2_1_/*n* space group and comprises the **[**Mn_3_(O_2_CMe)_4_(py)_8_]^2+^ cation and two I_3_^−^ counteranions. The cation of **1** (Figure 1) consists of a linear array of three Mn^II^ ions coordinated by four acetate groups and eight terminal pyridine molecules. The oxidation states of the Mn ions were determined by BVS calculations (Table 3), charge considerations, and inspection of metric parameters. The central metal ion of the trinuclear unit (Mn1), which is located on a crystallographic inversion center, is ligated by four oxygen atoms from four different acetate ligands and two molecules of pyridine adopting a distorted octahedral coordination geometry. All four acetate ligands bridge two Mn ions with two of them operating in the common *syn-syn*-*η* ^1^ : *η* ^1^ : *μ*_2_ fashion, whereas the other two function in the less common monoatomically bridging *η* ^2^ : *η* ^1^ : *μ*_2_ mode. The above mentioned carboxylate bridging modes have also been observed in several other linear trinuclear manganese (II) complexes [10–23]. However, in most linear Mn^II^_3_ complexes each pair of Mn^II^ ions is held together by at least three bridging ligands, whereas in **1** the neighboring Mn ions are connected through two bridging ligands only. One exception in this situation is the compound [Mn_3_(O_2_CMe)_6_(H_2_O)(phen)_2_] where one pair of Mn ions is linked through two acetate ligands, whereas the second one is held together by three bridging MeCOO^−^ ligands [16]. The consequence of the presence of less bridging ligands in **1** is the larger Mn⋯Mn separation (3.799 (2) Å) compared to the values observed in other linear trinuclear Mn^II^ complexes which are within the range of 3.2–3.7 Å [10–23]. The observed separation of 3.799 Å is slightly smaller than that (3.868 (4) Å) between the Mn ions bridged by two acetate ligands in [Mn_3_(O_2_CMe)_6_(H_2_O)(phen)_2_]. However, the Mn⋯Mn distance in the other pair of Mn ions of the latter is significantly shorter (3.489 Å) and thus the average Mn⋯Mn separation falls within the range observed for the other linear trinuclear Mn^II^ complexes.

The distorted octahedral coordination environment around each terminal metal ion (Mn2) is completed by three pyridine molecules. The Mn2N_3_O_3_ octahedron is significantly distorted, with the main distortion arising from the acute O3–Mn2–O4 angle (58.24 (7)°). The Mn1N_2_O_4_ octahedron is almost perfect. All Mn–N and Mn–O bond lengths of the two crystallographically independent manganese ions are within the expected range for octahedral high-spin Mn^II^ complexes.

**Figure 2:**
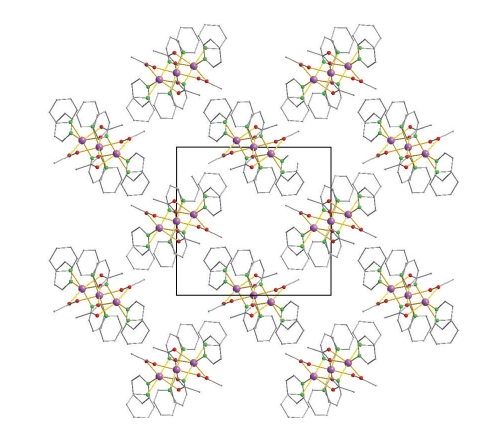
A representation of the packing of complex **1**. Mn, purple; O, red; C, grey. H atoms are omitted for clarity.

A close examination of the packing of **1 **revealed that the trinuclear molecules are nearly perpendicular to each other (Figure 2) and there are no significant hydrogen bonding interactions between neighboring units of **1**.

### 3.3. Magnetic Properties

**Figure 3:**
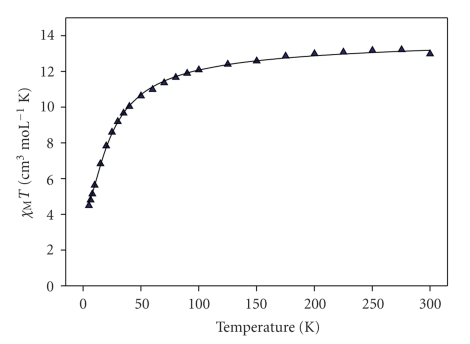
Plot of *χ*_*M*_ *T* versus *T* for **1**. The solid line is the fit of the experimental data; see the text for the fit parameters.

Solid-state dc magnetic susceptibility studies were performed on a powdered crystalline sample of **1** in a 0.1 T field and in the 5.0–300 K temperature range. The obtained data are plotted as *χ*_*M*_ *T* versus *T* in Figure 3. 

The *χ*_*M*_ *T* product at 300 K for **1** is 12.98 cm^3^ mol^−1^ K, slightly smaller than the value expected for three Mn^II^ (*S* = 5/2) noninteracting ions (13.125 cm^3^ mol^−1^ K, *g* = 2) indicating the existence of antiferromagnetic exchange interactions. This is corroborated by the continuous decrease of *χ*_*M*_ *T* upon cooling down to 10.63 cm^3^ mol^−1^ K at ~50 K. Below that temperature, the decrease is more abrupt, with *χ*_*M*_ *T* reaching a value of 4.49 cm^3^ mol^−1^ K at 5 K. The 5 K *χ*_*M*_ *T* value is very close to the spin - only (*g* = 2) value of 4.375 cm^3^ mol^−1^ K for a spin ground state *S* = 5/2. These results are indicative of antiferromagnetic exchange interactions between the Mn ions of **1** that lead to a spin ground state of *S* = 5/2.

 The magnetic susceptibility was simulated taking into account only one isotropic intracluster magnetic interaction, *J*, between Mn1 and Mn2 centers since the exchange interaction between the terminal Mn ions of **1** and also of most of the known linear Mn^II^_3_ complexes is negligible (*J*′ = 0) [10, 11, 15, 16] because of the large Mn⋯Mn separation (for **1** Mn2⋯Mn2′ = 7.598(1) Å). Application of the van Vleck equation [41] to the Kambe's vector coupling scheme [42] allows the determination of a theoretical *χ*_*M*_ versus *T* expression for **1** from the following Hamiltonian:

(2) $$H=-2J\left({\hat{S}}_{1}{\hat{S}}_{2}+{\hat{S}}_{1}{\hat{S}}_{2^{\prime }}\right),$$

using the numbering scheme of Figure 1, where *S*_1_ = *S*_2_ = *S*_2′_ = 5/2. This expression was used to fit the experimental data giving *J* = −1.50 K and *g* = 2.00 (solid line, Figure 3). A temperature-independent paramagnetism (TIP) term was held constant at 600 × 10^−6^ cm^3^ mol^−1^ K.

The obtained *J* value is smaller than values reported in the literature for other linear Mn^II^_3_ clusters with three bridging ligands per manganese pair which in most cases range from ~−2.5 to ~−7 K [18]. This behaviour could be rationalized on the basis of the existence of only two bridging ligands per manganese pair and larger Mn⋯Mn separations in **1** as was discussed in detail above (description of the structures). There are, however, examples of linear Mn^II^_3_ clusters with *J* values comparable to that of **1**, such as [Mn_3_(L^1^)_2_(*μ*-O_2_CMe)_4_] · 2Et_2_O (HL^1^ = (1-hydroxy-4-nitrobenzyl)((2-pyridyl)methyl)((1-methylimidazol-2-yl)methyl)amine) (*J* = −1.7 K) [21].

## 4. Conclusions

A new linear trinuclear manganese(II) complex [Mn_3_(O_2_CMe)_4_(py)_8_]^2+^ cocrystallizing with I_3_^−^ [**1**] and ClO_4_^−^ [**2**] has been synthesized serendipitously. Compound **1** was prepared in an attempt to oxidize [Mn(O_2_CMe)_2_] · 4H_2_O with I_2_ in MeCOOH/py, whereas compound **2** was initially isolated during our investigations on reactions of [Mn_3_O(O_2_CMe)_6_(py)_3_] · py with Mn(ClO_4_)_2_ · 6H_2_O in the presence of pdH_2_ in MeCN/py and was resynthesized in lower yield without adding pdH_2_ in the reaction mixture. Although several linear trinuclear Mn^II^ complexes have been prepared and studied, the cation of **1** and **2** has several novel structural features including: (i) different type of ligation since **1** and **2** are the first examples of linear trinuclear Mn clusters with only acetate and pyridine ligands and (ii) different number of bridging ligands between each pair of Mn^II^ ions, since **1** and **2** are rare examples of linear Mn^II^_3_ clusters with only two bridging ligands linking each pair of Mn^II^ ions. Variable temperature dc magnetic susceptibility studies revealed the existence of antiferomagnetic interactions between the Mn ions of **1** resulting in an *S*_*T*_ = 5/2 spin ground state.

## Figures and Tables

**Table 1:** Crystallographic data for complexes **1** and **2**.

	** 1**	** 2**
Formula^(a)^	C_48_H_52_Mn_3_N_8_O_8_I_6_	C_48_H_52_Mn_3_N_8_O_16_Cl_2_
*M*_*w*_	1795.20	1232.70
Crystal System	Monoclinic	Monoclinic
Space group	P2_1_/n	P2_1_/n
*a*/Å	15.2694(7)	21.7552(5)
*b*/Å	13.8883(4)	11.0081(2)
*c*/Å	15.2919(6)	23.4535(4)
*β*/°	109.041(5)	107.917(2)
*V*/Å^3^	3065.5(2)	5344.3(2)
*Z*	2	4
T/K	100(2)	100(2)
*λ* ^(b)^, Å	0.71073	0.71073
*D*_*c*_, g/cm^−3^	1.945	1.532
*μ* (Mo Ka)/mm^−1^	3.682	0.874
Refl. collected/unique (*R*_int _)	23443/7336 (0.0455)	34188/9376 (0.0733)
Obs. refl. [*I* > 2*σ*(*I*)].	5655	4469
*R*1 %^(c)^	0.0285	0.0382
*w* *R*2^(d)^	0.0640	0.0636
Goodness of fit on *F* ^2^	0.952	0.736
Largest diff. peak/hole/e^−^/Å^−3^	1.172/−1.043	0.366/−0.298

^(a)^Including counteranions. ^(b)^Graphite monochromator. ^(c)^ *R*1 = Σ||*F* *ο* | −|*F* *c*||/Σ | *F* *ο*|. ^(d)^ *w* *R*2 = [Σ[*w*(*F*_*o*_^2^−*F*_*c*_^2^)^2^]/Σ[*w* *F*_*o*_^2^)^2^]]^1/2^, *w* = 1/[*σ* ^2^(*F*_*o*_^2^) + (*m* · *p*)^2^ + *n* · *p*], *p* = [max (*F*_*o*_^2^, 0) + 2*F*_*c*_^2^]/3, and *m* and *n* are constants.

**Table 2:** Selected interatomic distances (Å) and angles for complex **1**.

Bond Distances (Å)					
	Mn1⋯Mn2			3.799(2)	
	Mn1–O1			2.154(2)	
	Mn1–O3			2.196(2)	
	Mn1–N1			2.247(2)	
	Mn2–O2			2.093(2)	
	Mn2–O3			2.234(2)	
	Mn2–N3			2.235(2)	
	Mn2–O4			2.276(2)	
	Mn2–N2			2.288(2)	
	Mn2–N4			2.295(2)	

Bond Angles (°)					

O1–Mn1–O1		180.0	O2–Mn2–O3		103.82(7)
O1–Mn1–O3		90.90(7)	O2–Mn2–N3		108.98(7)
O1′–Mn1–O3		89.10(7)	O3–Mn2–N3		146.90(7)
O3–Mn1–O3′		180.0	N3–Mn2–O4		89.50(7)
O1–Mn1–N1		90.57(7)	O2–Mn2–N2		90.28(8)
O1′–Mn1–N1		89.43(7)	O3–Mn2–N2		96.50(7)
O3–Mn1–N1		87.20(7)	N3–Mn2–N2		87.73(8)
O3′–Mn1–N1		92.80(7)	O4–Mn2–N2		86.28(7)
O1–Mn1–N1		90.57(7)	O3–Mn2–N4		89.30(7)
N1–Mn1–N1′		180.0(2)	N2–Mn2–N4		173.85(8)
			Mn1–O3–Mn2		118.07(7)

**Table 3:** Bond valence sum (BVS)^(a,b)^ calculations for complexes **1** and **2**.

	*Complex **1***			*Complex **2***		
	Mn^II^	Mn^III^	Mn^IV^	Mn^II^	Mn^III^	Mn^IV^
Mn1	2.00	1.87	1.90	1.92	1.81	1.81
Mn2	1.90	1.79	1.80	2.05	1.91	1.95
				1.91	1.80	1.81

^(a)^The underlined value is the one closest to the charge for which it was calculated. ^(b)^The oxidation state is the nearest whole number to the underlined value.
